# Development and Validation of HPLC-DAD Method for Simultaneous Determination of Seven Food Additives and Caffeine in Powdered Drinks

**DOI:** 10.3390/foods9081119

**Published:** 2020-08-13

**Authors:** Imanulkhan, Widiastuti Setyaningsih, Abdul Rohman, Miguel Palma

**Affiliations:** 1 Department of Food and Agricultural Product Technology, Faculty of Agricultural Technology, Gadjah Mada University, Jalan Flora, Bulaksumur, Yogyakarta 55281, Indonesia; imanulkhan@mail.ugm.ac.id; 2 National Agency of Drug and Food Control, District of Kupang, R.A. Kartini street, Kota Baru, Kelapa Lima, Kupang 85228, East Nusa Tenggara, Indonesia; 3 Department of Pharmaceutical Chemistry, Faculty of Pharmacy, Gadjah Mada University, Sekip Utara, Bulaksumur, Yogyakarta 55281, Indonesia; abdul_kimfar@ugm.ac.id; 4 Department of Analytical Chemistry, Faculty of Sciences, IVAGRO, University of Cadiz, Campus de Excelencia Internacional Agroalimentario (CeiA3), Campus del Rio San Pedro, Puerto Real, 11510 Cádiz, Spain; miguel.palma@uca.es

**Keywords:** food additives, HPLC, multi-response optimization, Box–Behnken Design, powdered drinks

## Abstract

The usage of food additives must respect the general legislation in force in the country and requires a reliable analytical method for surveillance. This research aimed to develop a high-performance liquid chromatography with diode array detection (HPLC-DAD) method for the simultaneous determination of seven food additives and caffeine in powdered drinks. Three factors likely to affect the chromatographic separation, namely, mobile phase composition at the beginning (*x*_1_, 0–10% of the amount of methanol in the phosphate buffer) and the end (*x*_2_, 60–100% of the amount of methanol in the phosphate buffer) of the gradient program and pH (*x*_3_, 3–7), were evaluated with the aid of a Box–Behnken Design (BBD). Subsequently, multi-response optimizations for chromatographic resolutions (*Rs*) and analysis time were performed using the response surface methodology (RSM) in conjunction with the desirability function (DF). Complete separation (*Rs* > 1.5) of seven food additives and caffeine was achieved in less than 16 min by applying 8.5% methanol in the phosphate buffer at the beginning and 90% at the end of the gradient program, in pH 6.7. The developed method was validated with low limits of detection (ranging from 1.16 mg kg^−1^ (sodium saccharin) to 3.00 mg kg^−1^ (acesulfame potassium)), low limits of quantification (ranging from 3.86 mg kg^−1^ (sodium saccharin) to 10.02 mg kg^−1^ (acesulfame potassium)), high precision (CV < 4%), and high accuracy (recoveries from 95 to 101% at 80, 100, and 120% of the target concentration). The method was successfully used to assess the seven food additives and caffeine in commercially available powdered drinks.

## 1. Introduction

Powdered drinks, apart from having a longer self-life, are more reasonably priced than juices or soft drinks [1]. This fact supports the growth of product innovations employing food additives to offer a vast array of instant flavored drinks in the market. Food additives play a significant role in the improvement of food product appearance and taste as well as prevent alteration and deterioration of the powdered drink products [2]. However, the usage of food additives must respect the general legislation in force in the country to ensure product safety. In Indonesia, for instance, all food additives must be listed on the product labels and are kept under surveillance by the National Agency of Drug and Food Control (NADFC) [3]. Therefore, a reliable analytical method to check the composition of additives used and their levels in food products are required for surveillance.

On the basis of the NADFC database, some of the most common available additives to prepare commercial powdered drinks include a group of sweeteners, colorants, preservatives, and flavor enhancers [4,5,6,7]. The most commonly used analytical techniques for the additives determination in foods include spectrophotometry [8] in addition to liquid [9,10,11,12] and gas [13] chromatography. Although several developed methods are available, the existing methods are mainly focused on multi-parallel analysis [14,15,16]. The multi-parallel analysis approaches are inefficient as they are time-consuming and require more chemical reagents, thus leads to a higher economic expense. Therefore, a method that determines all relevant additives in particular food products in a single analysis would be convenient, primarily to support rapid surveillance in the market. However, actual multi-analyte methods for additives in commercial powdered drinks are still limited. The existing analytical methods merely facilitate the determination of some additives rather than the complete composition of the samples [17,18,19].

The analytical technique of choice in this study was high-performance liquid chromatography (HPLC) coupled with a diode array detector (DAD). The development of the HPLC method is mainly related to the optimization of the elution power of the mobile phases for full separation. This optimization can be performed by checking the programmed gradient elution [20,21] for several compounds. Additionally, the pH of the mobile phases is also an important factor for the separation results [21]. The aforementioned separation factors greatly affect the resulting resolution of the adjacent peaks in the chromatogram. Apart from acceptable resolutions, the HPLC condition can be optimized to achieve a reasonable analysis time [22,23,24]. Hence, in this study, the effects of the gradient program and the pH of mobile phases were optimized for a complete separation of eight typical ingredients (acesulfame potassium, benzoate acid, sorbic acid, sodium saccharin, tartrazine, sunset yellow, caffeine, and aspartame) commonly used in commercial powdered drinks.

Response surface methodology (RSM) is considered effective at optimizing a number of variables affecting the separation performance of the chromatographic method [25]. Although some experimental designs are available to couple with the RSM, a Box–Behnken Design (BBD) provides fewer runs for three experimental variables [26]. Because the optimization of the chromatographic method involves several dependent variables such as resolutions and analysis time, it requires simultaneous evaluation for the response [27]. Therefore multi-response optimization using the desirability function is an excellent option to obtain a solution for simultaneous optimization [26]. Setyaningsih et al. [25] have successfully used the aforementioned experimental design for multi-response optimization of UHPLC conditions to achieve a complete separation for the simultaneous determination of 20 studied compounds.

This study aimed to optimize the HPLC-DAD method for the simultaneous separation of seven food additives and caffeine in powdered drinks using a BBD in conjunction with a desirability function. As part of the method’s validation process, the optimized method was applied to a number of commercial instant drink flavored powders.

## 2. Materials and Methods

### 2.1. Chemical and Reagents

Potassium dihydrogen phosphate, dipotassium hydrogen phosphate, phosphoric acid, potassium hydroxide, and HPLC-grade methanol for the chromatographic analyses were obtained from E. Merck (Darmstadt, Germany). Aqua bidest was obtained from PT Ikapharmindo Putramas. Reference standards of acesulfame potassium (ACE; B0214023; purity 100.41%), benzoic acid (BEN; B0114244; purity 99.90%), sorbic acid (SOR; B0315017; purity 99.82%), sodium saccharin (SAC; B0216303; purity 101.96%), tartrazine (TAR; 110397; purity 96.96%), caffeine (CAF; 413017; purity 99.79%), sunset yellow FCF (SUN; B0215284; purity 97.97%), and aspartame (ASP; B0216366; purity 98.56%) were obtained from the National Agency of Drug and Food Control of the Republic of Indonesia. Standard stock solutions of the studied food additives were prepared in a mixture of water and methanol 50:50 (*v*/*v*), having a concentration of 1000 mg L^−1^. Standard working solutions were prepared by dissolving the standard stock solutions using aqua bidest, resulting in concentrations from 0.5 to 50 mg L^−1^. Both solutions were stored in the refrigerator at 8 °C.

### 2.2. Chromatographic Method

The chromatographic separation was carried out on a Shimadzu HPLC system (Kyoto, Japan) equipped with a binary pump (LC-20AD), auto-sampler (SIL-HTC, Shimadzu, Japan), and UV-Vis SPD M-20A diode array detector (DAD). The detector was set for compound identification using a three-dimensional (3D) scan mode in the wavelength range from 190 to 350 nm (Supplementary Figure S1).

According to the UV spectra obtained with a DAD, the maximum absorptions of the compounds under investigation were in the range from 196 nm (CAF and SUN) to 252 nm (SOR), whilst all of the studied compounds could produce signals at 210 nm. Hence, during the optimization, peak integrations were performed at 210 nm to be able to observe all peaks and to calculate the resolution. However, a fixed wavelength of the UV absorption maxima using a two-dimensional (2D) scan mode was chosen for method validation and quantification for the respective compounds, i.e., 200 nm for SAC, TAR, CAF, and ASP; 225 nm for ACE, BEN, and SOR; and 235 nm for SUN. The studied compounds in the samples were identified by comparing their retention times and spectra properties with the standards.

The separation of seven food additives and caffeine in a 20 μL injected sample was performed on a reverse-phase C_18_ column Shim-Pac GIST Shimadzu (150 mm, 4.6 mm, 5 μm) at a column temperature of 30 °C. The mobile phases consisted of phase A (phosphate buffer) and phase B (methanol) at a flow rate of 1 mL min^−1^. The pH value of phase A was fixed following the experimental design. The linear gradient program was set for 30 min. Subsequently, the column was cleaned for 5 min (100% mobile phase B) and equilibrated for 7 min (2 min running with a linear gradient from 100% mobile phase B to the initial mobile phase composition of the next injection according to the BBD and 5 min running with that mobile phase composition) before the analysis. LC Solution Software version 1.2 SP1 was used to control the hardware and process the data.

### 2.3. Sample Preparation

Nine products of commercial flavored powdered drinks were purchased from supermarkets in Yogyakarta, Indonesia. Samples were weighed accurately (0.5 g) and diluted into 100 mL aqua bidest. The solution was then filtered with a 0.45 µm nylon filter before injecting 20 µL of the solution into the HPLC system.

### 2.4. Box–Behnken Design and Data Analysis

**Figure 1:**
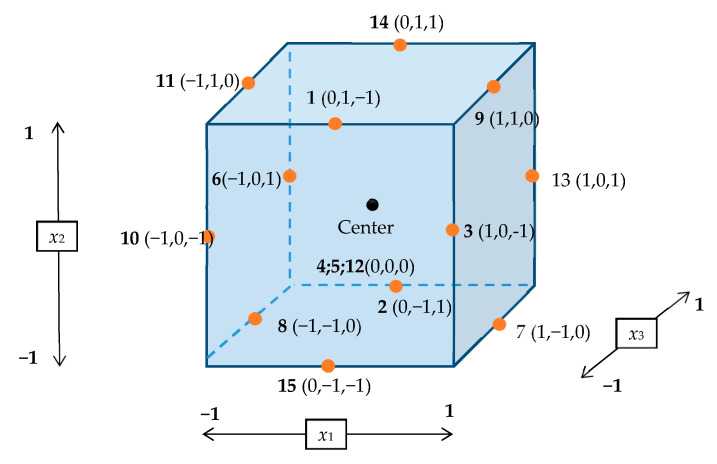
A 15-run experiment of the Box–Behnken Design (BBD).

Prior to the optimization, the selection of the factors influencing the separation of compounds by the HPLC-DAD was conducted through a comprehensive evaluation based on the previous reports [17,18,22,23,28,29,30]. The most common experimental variables that most likely affect the separation of seven food additives and caffeine by HPLC-DAD are mobile phase composition at the gradient start (%B _initial_, *x*_1_) and the end of gradient elution (%B _end_, *x*_2_) and the pH of the mobile phase (pH, *x*_3_). A Box–Behnken Design was constructed on the basis of those three experimental variables, three levels, and three central points, resulting in a 15-run experiment (Figure 1). The three levels were priorly coded as −1, 0, and 1 (low, center, and high level, respectively) to ascertain a more even response [31], as shown in Table 1.

The responses considered for the optimization were (i) the resolution (*Rs*) of chromatographic peaks and (ii) the analysis run time. The retention time of the final peak in the chromatogram was regarded as the response for the analysis run time, while the peak resolution (*Rs*) was defined as a value describing the separation of two adjacent peaks in terms of their average baseline peak width. Resolution values for all peaks in the chromatogram were used as responses. Hence, by definition, the resolution was calculated using the following equation:

(1) $$Rs=\frac{t_{2}-t_{1}}{\frac{1}{2}\;\left(w_{1}+w_{2}\right)}$$

where *Rs* is the peak resolution; *t*_1_ and *t*_2_ are the retention times of the first and second peaks, respectively; *w*_1_ and *w*_2_ are the corresponding widths at the bases of the pair of adjacent peaks.

Once the responses from the BBD had been acquired, a mathematical model was built for each response. The model required to be fitted with a second-order polynomial function. The following general equation was used for this purpose:

(2) $$y=\beta_{0}+\overset{k}{\underset{i=1}{\sum }}\beta_{i}x_{i}+\overset{k}{\underset{i=1}{\sum }}\beta_{ii}x_{i}^{2}+\overset{k}{\underset{i=1}{\sum }}\overset{k}{\underset{j=1,\;j\neq i}{\sum }}\beta_{ij}x_{i}x_{j}$$

where *x_i_*, *x_j_*, …, *x_k_* are the variables that influence the response *y*; *β*_0_, *β*_ii_ (*i* = 1, 2, …, *k*), and *β*_ij_ (*i* = 1, 2, …, *k*) are unknown parameters. The *β* coefficients were obtained by the partial least squares method. Generally, only second-order interactions are taken into account to build models because higher-order interactions are usually not significant and may be confused with the main effects.

The desirability function using STATGRAPHICS Centurion XVI (Statpoint Technologies Inc., Warrenton, VA, USA) was used to find the optimum chromatographic conditions.

### 2.5. System Suitability Test

System suitability testing (SST) is required to check and ensure the on-going performance of analytical systems and methods. SST was assessed based on the precision of an HPLC condition for the intended analysis. Hence, SST was carried out by injecting a mixture solution of seven food additives and caffeine standards, each at concentrations of 10 mg L^−1^ with six replicates. The SST parameters that were evaluated include retention time (*t*) and peak area. The coefficient of variation (%CV) of each set of parameters (retention time and peak area) should be less than 2% [32].

### 2.6. Method Validation

The new method for the simultaneous separation of seven food additives and caffeine in the powdered drinks was validated based on the recommendations by ISO 17025 and the International Conference on Harmonization (ICH) Guideline Q2 (R1) [33,34]. The detection and qualification limits, the range of linearity, and the precisions of the method were determined. A series of solutions were prepared using the standard reference of the eight studied analytes to reach concentration from 1 to 50 mg L^−1^. Once the regression analysis was calculated, the linearity was then measured within the studied range to confirm that the test results obtained by the method are proportional to the concentration of the analytes. Based on the regression data of slope and standard error, the limit of detection (LOD) and limit of quantification (LOQ) were estimated.

The accuracy of the method was evaluated by a standard addition (spiking) technique. Recovery values of the standard analytes spiked into the solid sample at concentration levels of 80%, 100%, and 120% of the target concentration were calculated. The target concentrations ranged from 5 (SUN) to 500 mg Kg^−1^ (ASP). A high accuracy was determined by recovery values ranging from 80% to 110%.

Repeatability (intraday) and intermediate precision (interday) for the chromatographic results were used to indicate the precisions of the developed method. Six independent analyses on the same day of the same real samples spiked with 10 mg L^−1^ of the corresponding analyte were used to establish the value for the repeatability. A spiked real sample was used for the usual matrix. In comparison, three independent analyses on three consecutive days of the same samples spiked with 10 mg L^−1^ of the corresponding analyte were used to determine the intermediate precision. Coefficients of variation (CVs) of the retention time and peak area were used to express the precision. According to the Association of Official Analytical Collaboration (AOAC) International Manual for the Peer-Verified Methods Program, the acceptable CV limit is ±10% [35].

## 3. Results

### 3.1. Data Acquisition for the Responses

A mixture of seven standards of the food additives and caffein was injected into the HPLC system in accordance with the BBD. The analytes were eluted in the following order of increasing retention time: 1. ACE; 2. BEN; 3. SOR; 4. SAC; 5. TAR; 6. CAF; 7. SUN; 8. ASP. Hereafter, these order numbers indicate the corresponding compounds, as cited in the peak resolution. *Rs*1–2 means resolution between ACE and BEN, and so forth. The resulting *Rs* values of the peaks of the eight studied analytes are presented in Table 2. Complete separation was found for *Rs* equal to 1.5 or higher. However, an *Rs* with a value of 1.0 was acceptable because it produced 98% separation. Values below 1.0 were considered to give poor separation [31]. Henceforth, specific *Rs* showing some values less than 1.0 were included in the optimization process with the target of maximizing the values reaching full separation.

In addition to *Rs*, the total time to elute the last peak, indicating the analysis time, was also included in the optimization as the response because it should be kept as low as possible. The analysis run time ranged from 14.7 to 25.4 min. Hence, by counting the number of *Rs* values that that were lower than 1.0 and the analysis run time, the total number of responses for the optimization was eight.

### 3.2. Optimization of the Separation Method

Prior to the use of the multi-response optimization (MRO), the response surface methodology (RSM) data were calculated to generate a model for each response through regression analysis. As a result, eight models were constructed for seven *Rs* values and analysis time. Each model demonstrates the empirical relationship between the three studied variables and the responses. The calculated regression coefficients are given in Table 3, and these results explain the effects of the studied variables and interactions.

Analysis of variance (ANOVA) was used to assess the variability in responses and separate the responses into pieces for each of the effects. It was then used tested the statistical significance of each effect by comparing the mean square against an estimate of the experimental error. Variables with a *p* value coefficient of less than 0.05 were defined as having a significant effect on the corresponding response. The regression coefficients were then used for a final predictive equation of RSM for *Rs* and analysis time by using only significant variables.

The significant variables for each response were varied. The positive effect of %B _initial_ (*x*_1_) was important for *Rs*6–7, while *Rs*4–5 and analysis time were negatively affected by %B _end_ (*x*_2_). The mobile phase composition defines the affinity of analytes to the mobile phase. A stronger affinity between analytes with the mobile phase could lead to a higher elution power. %B _end_ indicates the use of the organic methanol in the gradient program. With lower %B _end_, the elution power is reduced, resulting in better separation for SAC-TAR (*Rs*4–5). Similar findings were reported for the use of methanol as eluent for food additives separation by chromatographic techniques [16,36,37].

Additionally, according to the ANOVA results, both main and quadratic effects of pH significantly contributed (*p* < 0.05) to the separation for most peaks (*Rs*1–2, *Rs*2–3, *Rs*4–5, *Rs*5–6, and *Rs*6–7). The importance of pH in the mobile phase was considered to improve the selectivity of the chromatographic method [18,38]. The positive linear term (*x*_3_) indicated that the higher the pH, the higher the resulting resolution. The increase in pH facilitated the ionization process of acidic analytes in the polar mobile phase, which increased the solubility of the analytes. In contrast, the buffer solution with a higher pH hardly eluted the basic analytes because they were less ionized [35]. Hence, a complete separation of adjacent peaks consisting of the acidic and less acidic analytes, e.g., BEN-SOR (*Rs*2–3) and TAR-CAF (*Rs*5–6), can be achieved by increasing the pH. Additionally, a significant effect of pH on the resolution is related to the available chemical forms for the compounds during the elution. If the working pH is near the pKa, more than one chemical form could be found; therefore, broad peaks or even more than one peak would be produced from one compound.

Using only the significant variables, models were built for predicting each response. The models were validated by the coefficient of determination (*R*^2^) that ranged from 0.7414 (*Rs*3–4) to 0.9745 (*Rs*1–2). In addition, the mean absolute error (MAE) ranged from 0.0830 (*Rs*7–8) to 0.5456 (analysis time). Because of the high values of *R*^2^ and low error, the models can be used for reliable prediction in multi-response optimization (MRO).

The MRO was employed to optimize the eight responses simultaneously. The desirability function *d*(*y*) was then built based on the values obtained for each optimized response. The MRO approach assumes the response values equal to (*y*) can be modeled through the *d*(*y*), where the desirability ranges from 0 to 1.

**Figure 2:**
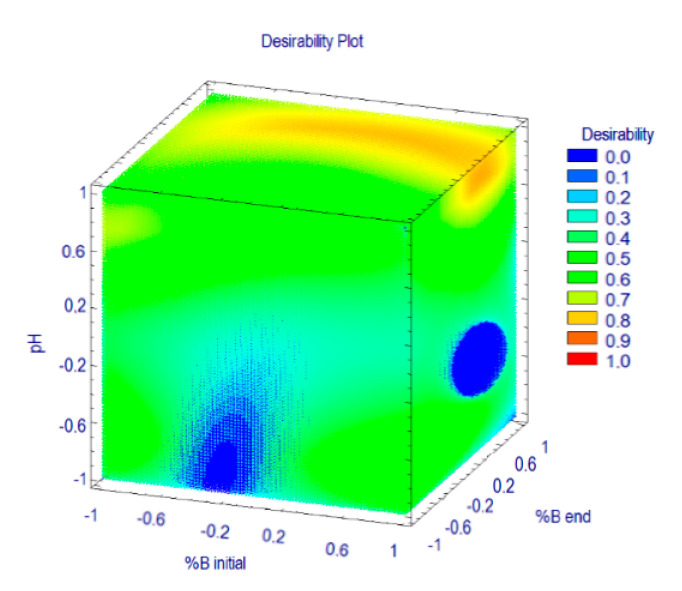
3D contour plot showing interactive effects of % B *start* (*x*_1_), % B *end* (*x*_2_), and pH (*x*_3_) over desirability for the HPLC-DAD method.

**Figure 3:**
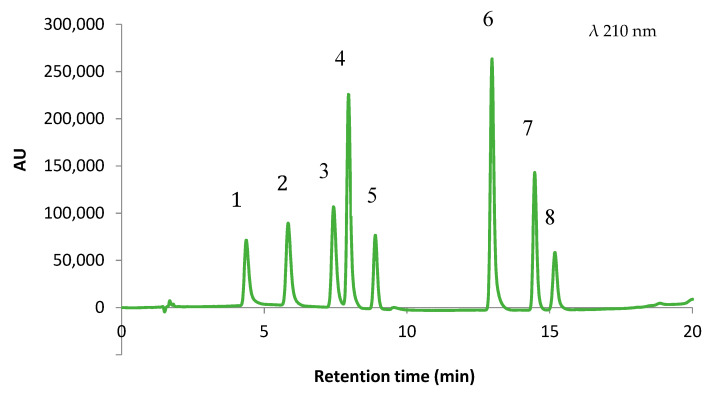
Chromatogram of seven food additives and caffeine using the optimized method for the HPLC-DAD (1. ACE; 2. BEN; 3. SOR; 4. SAC; 5. TAR; 6. CAF; 7. SUN; 8. ASP).

In this optimization by the desirability function, the response of analysis time was set as less important (the lowest importance with impact coefficient of 1) than *Rs* (the default importance with impact coefficient of 3). These settings were due to the range of the analysis run time being adequate for a fast HPLC method. In the MRO, the importance of the responses for computational analysis was indicated by the impact coefficient given to the responses. By using the STATGRAPHICS Centurion XVI, values of the impact coefficients can be set from 1 to 5. On the basis of these settings, a 3D contour plot was built for MRO (Figure 2). It can be seen that the maximum value for the desirability function can be found at %B _initial_ = 0.69 (*x*_1_; 8.5%), %B _end_ = 0.49 (*x*_2_; 90.0%), and pH = 0.88 (*x*_3_, 6.7). Using those conditions, a complete separation was achieved in roughly 15 min (Figure 3).

The optimum separation condition was then confirmed by performing the analysis of the studied analytes in a mixture of standard solution to check the selectivity of the method. The resulting chromatogram by the optimum condition suggested by the MRO is presented in Figure 3.

The proposed method could completely separate the eight analytes, as indicated by the resolution values higher than 1.5. The resulting resolution values by the optimized method ranged between 1.51 (*Rs*3–4) and 9.56 (*Rs*5–6). Additionally, this separation method could be considered as a rapid chromatographic method because the analysis time was less than 16 min. This report is faster than previous studies since it provides faster analysis time to separate similar or even higher numbers of analytes yet with complete resolutions [18,22].

### 3.3. Validation of the Separation Method

Validation of the analytical method is a procedure to prove whether an analysis method meets the specified requirements so that the results of the analysis can be justified. In this study, the system suitability test (SST) was also assessed prior to evaluating the other validation parameters. Table 4 summarizes the results of the HPLC-DAD method validation.

The results showed that the system suitability test (%CV) values of each set of parameters were less than 2%, indicating the high precision of the HPLC-DAD system. The regression of the calibration curves for all analytes provided a high coefficient of determination (*R*^2^), 0.9991 or more in the studied range 1 to 50 mg L^−1^. The LOD values ranged from 1.16 mg kg^−1^ (SAC) to 3.00 mg kg^−1^ (ACE), while the LOQ values ranged from 3.86 mg kg^−1^ (SAC) to 10.02 mg kg^−1^ (ACE). The low LOD and LOQ facilitate a reliable detection and quantification of seven food additives and caffeine with very low concentration in instant flavored drinks.

Recovery represents a measure that indicates the degree of closeness of the analysis results with the actual level of the analyte. The standard addition experiments resulted in recoveries that ranged from 95.30% to 101.41%. Providing recoveries near to 100%, the results indicate the confidence of the method to measure the level of the studied compounds in the sample.

Intraday and interday precision were calculated to establish the precision of the method. The CVs for intraday precision of the retention time and the signal of the area of the studied analytes, on average, were 0.28% and 2.14%, respectively, while the intermediate precisions were 0.25% and 2.53%, respectively. It was also observed that CAF and ASP have the highest precisions. Based on the acceptance values suggested by the AOAC through the International Manual for the Peer-Verified Methods Program, the proposed method has been validated due to the high precisions [30].

The validated HPLC-DAD method was then applied for the simultaneous determination of seven food additives and caffeine in nine popular powdered drinks in the market. The chosen samples consisted of four powdered drinks with fruity flavor (samples 01 to 04), one powdered drink with sweet tamarin flavor (sample 05), and the rest of the samples (samples 06 to 09) were powdered drinks based on tea. The composition of the relevant ingredients of the samples is given in Table 5a.

Applying the HPLC-DAD method to the nine powder drink samples, seven food additives and caffeine were successfully identified and quantified (Table 5b). The method detected and provided the levels of the analytes mentioned on the products’ labels. Although CAF was not listed as an ingredient in samples 06 to 09, it was found to be present in the products. Samples 06 to 09 consisted of tea, and this information was claimed in the label as an ingredient, which provided the natural CAF to the product.

National and international regulations are applied to control the use of food additives in powdered drinks. In Indonesia, the actual regulation is referred to as the National Agency of Drug and Food Control (NADFC) guidance. The established guidance rules for the studied additives in powdered drink products are as follows: ACE, max. 600 mg kg^−1^; BEN, max. 600 mg kg^−1^; SOR, max. 1000 mg kg^−1^; SAC, max. 120 mg kg^−1^; TAR, max. 300 mg kg^−1^; CAF, max 250 mg kg^−1^; SUN, max. 300 mg kg^−1^, and ASP, max. 600 mg kg^−1^. The amount of food additives in the nine samples assessed here were all below the limits defined by the NADFC legislation for food additives in powdered drinks [3,5,6,7].

## 4. Conclusions

HPLC-DAD was developed and validated for the simultaneous determination and quantification of seven food additives and caffeine in powdered drinks. This method offers the advantage of using a short run time of 16 min for the separation of seven structurally related food additives and caffeine on the C_18_ column with a mobile phase consisting of buffer phosphate (pH 6.7) in water and methanol. Full separation of seven food additives and caffeine was achieved (*Rs* > 1.5) applying a mobile phase composition of 8.5% and 90% at the beginning and the end of the gradient program, respectively, and detection at a wavelength of 210 nm. Results from validation of the method proved satisfactory linearity, accuracy, and precision; therefore, we conclude that the method is suitable for routine quantification of the seven food additives and caffeine in powdered drinks available on the market.

## Figures and Tables

**Table 1:** Selected variables and their levels.

Variables	−1	0	+1
*x*_1_, %B _initial_	0	5	10
*x*_2_, %B _end_	60	80	100
*x*_3_, pH	3	5	7

**Table 2:** Observed responses of BBD by HPLC-DAD.

BBD Point	Resolution (*Rs*)							Analysis Time (min)
	*Rs*1–2	*Rs*2–3	*Rs*3–4	*Rs*4–5	*Rs*5–6	*Rs*6–7	*Rs*7–8	
1	FOP	FOP	1.2280	0.9515	0.6610	1.6360	0.7855	19.09
2	0.2555	1.1254	1.5789	1.5465	3.0378	2.7325	0.3874	21.18
3	FOP	FOP	1.5295	1.0145	0.5500	2.5180	1.0330	20.27
4	3.1026	1.2854	0.8438	2.6014	FOP	1.2804	0.8706	17.84
5	3.0046	1.2852	0.8660	2.6402	FOP	1.2772	0.8807	17.84
6	FOP	1.1549	1.6432	1.1495	3.1962	1.5370	0.9020	19.56
7	3.0033	1.9175	1.1825	3.2517	0.1641	1.1538	FOP	18.60
8	3.4380	1.2154	0.8799	2.4530	FOP	1.2758	0.9002	19.20
9	2.4517	0.9708	2.6548	1.7886	1.0280	FOP	0.9276	14.70
10	FOP	FOP	1.2460	1.4690	0.5300	2.3685	1.0175	22.76
11	3.2329	0.9794	2.1677	1.8457	1.2248	0.6605	0.9197	17.15
12	3.1698	1.3190	0.8337	2.5400	FOP	1.2681	0.8802	17.82
13	1.0052	1.4346	1.1752	1.3097	4.0203	2.2164	0.8106	16.67
14	0.6670	1.8968	1.7036	1.2585	3.4432	1.0378	1.1120	16.25
15	FOP	FOP	1.0430	1.2720	3.1895	3.1895	1.3860	25.40

Note: FOP, fully overlapped peaks.

**Table 3:** Regression coefficients and validation parameters for the model in the regression analysis.

Model Term	Regression Coefficients							
	*Rs*1–2	*Rs*2–3	*Rs*3–4	*Rs*4–5	*Rs*5–6	*Rs*6–7	*Rs*7–8	Analysis Time
constant	3.092	1.297	0.848	2.594	0.000	1.275	0.877	23.085
*x*_1_, %B _initial_	−0.026	0.122	0.076	0.056	0.098	**0.006**	−0.121	−0.620
*x*_2_,%B _end_	−0.043	−0.051	0.384	**−0.335**	−0.008	−0.627	0.134	**−3.039**
*x*_3_, pH	0.241	**0.701**	0.132	0.070	**1.096**	−0.274	−0.126	−0.617
*x*_1_ *x*_1_	−0.020	−0.067	0.442	−0.140	0.044	−0.246	−0.084	−0.672
*x*_1_ *x*_2_	−0.087	−0.178	0.046	−0.214	−0.097	−0.135	0.227	−0.774
*x*_1_ *x*_3_	0.251	0.070	−0.188	0.154	0.201	0.132	−0.027	−0.025
*x*_2_ *x*_2_	−0.041	0.041	0.432	−0.119	0.553	−0.257	−0.107	0.208
*x*_2_ *x*_3_	0.103	0.193	−0.015	0.008	0.733	−0.035	**0.331**	0.085
*x*_3_ *x*_3_	**−2.821**	**−0.582**	0.109	**−1.218**	**2.030**	**1.131**	0.147	0.366
**Model Validation**								
*R* ^2^	0.9743	0.9153	0.7414	0.9370	0.8794	0.9401	0.8679	0.9375
*R* ^2^_(adjustedford.f)_	0.9281	0.7629	0.2758	0.8236	0.6624	0.8324	0.6301	0.8260
SE	0.4005	0.3234	0.4443	0.3008	0.8749	0.3461	0.1908	1.0064
MAE	0.1987	0.1477	0.1983	0.1392	0.3958	0.1577	0.0830	0.5456

Note: SE, standard error of estimation; MAE, mean absolute error; Values in bold red text indicate that the corresponding variables had a significant effect (*p* value < 0.05).

**Table 4:** Analytical properties for the validated HPLC-DAD method.

Analytes	SST (%CV)		Linear Equation	*R* ^2^	Limits (mg kg^−1^)		Recovery (%)			Intraday, %CV (*n* = 9)		Interday, %CV (*n* = 3 × 3)	
	*t*	Area			LOD	LOQ	80%	100%	120%	*t*	Area	*t*	Area
ACE	0.56	0.60	*y* = 66,040.5*x* − 19359.2	0.9992	3.00	10.02	99.30	95.37	100.29	0.46	1.34	0.33	2.67
BEN	0.33	1.37	*y* = 78,686.4*x* − 21997.5	0.9994	2.16	7.21	101.41	97.87	96.28	0.40	2.77	0.36	3.03
SOR	0.24	0.75	*y* = 84,155.0*x* + 15085.0	0.9994	2.70	9.00	100.19	97.86	99.15	0.27	2.10	0.24	1.75
SAC	0.27	1.08	*y* = 169,869.9*x* − 28700.4	0.9998	1.16	3.86	96.90	98.75	97.23	0.30	3.02	0.29	4.06
TAR	0.33	1.08	*y* = 71,332.1*x* + 5665.2	0.9995	2.06	6.86	100.54	101.19	99.43	0.30	2.26	0.42	2.31
CAF	0.17	0.98	*y* = 122,490.4*x* + 27542.7	0.9997	1.93	6.43	99.32	96.11	97.11	0.14	1.29	0.10	2.58
SUN	0.24	0.24	*y* = 63,931.9*x* + 15471.2	0.9997	1.75	5.85	100.23	100.76	95.30	0.20	1.44	0.19	2.48
ASP	0.17	1.70	*y* = 55,293.4*x* − 31270.7	0.9991	2.98	9.95	98.68	98.31	101.74	0.14	2.94	0.08	1.85

Note: *y* is the peak area in the HPLC-DAD chromatogram; *x* is the corresponding analyte concentration in the injected sample; SST, system suitability test; %CV, coefficient of variation.

**Table 5:** (**a**) Relevant ingredients listed in the product label. (**b**) Real sample application of the validated HPLC-DAD method for seven additives and caffeine in powdered drinks.

**(a)**								
**Sample**	**ACE**	**BEN**	**SOR**	**SAC**	**TAR**	**CAF**	**SUN**	**ASP**
01	√	-	-	-	√	-	√	√
02	√	-	-	-	√	-	√	√
03	√	-	-	-	√	-	-	√
04	-	√	√	√	-	-	-	√
05	√	-	-	-	√	-	-	√
06	√	-	-	-	√	-	-	√
07	-	-	-	-	-	-	-	√
08	√	-	-	-	-	-	-	√
09	√	-	-	-	√	-	-	√
**(b)**								
**Sample**	**Concentration (mg kg^−1^)**							
	**ACE**	**BEN**	**SOR**	**SAC**	**TAR**	**CAF**	**SUN**	**ASP**
01	181.97 ± 0.45	ND	ND	ND	61.72 ± 0.14	ND	5.33 ± 0.44	446.96 ± 0.44
02	140.86 ± 0.56	ND	ND	ND	18.80 ± 0.13	ND	8.11 ± 0.01	495.24 ± 0.38
03	185.71 ± 0.72	ND	ND	ND	12.77 ± 0.06	ND	ND	156.75 ± 0.14
04	ND	77.87 ± 0.53	42.50 ± 0.13	117.97 ± 0.45	ND	ND	ND	476.01 ± 0.85
05	204.68 ± 0.87	ND	ND	ND	15.66 ± 0.15	ND	ND	163.25 ± 0.28
06	491.17 ± 0.72	ND	ND	ND	12.04 ± 0.32	31.45 ± 0.20	ND	188.11 ± 0.96
07	ND	ND	ND	ND	ND	94.62 ± 0.46	ND	274.82 ± 0.17
08	70.68 ± 0.50	ND	ND	ND	ND	66.17 ± 0.64	ND	437.57 ± 0.27
09	173.10 ± 0.73	ND	ND	ND	10.51 ± 0.06	22.06 ± 0.38	ND	258.76 ± 0.62

Note: acesulfame potassium (ACE), benzoate acid (BEN), sorbic acid (SOR), sodium saccharin (SAC), tartrazine (TAR), caffeine (CAF), sunset yellow FCF (SUN), and aspartame (ASP).“-” Not listed on the label as an ingredient, “√” Listed on the label as an ingredient, “ND” Not detected. The values are mean ± standard deviation of experiments performed in duplicate.

## Supplementary Materials

The following are available online at https://www.mdpi.com/2304-8158/9/8/1119/s1, Figure S1: Spectra of the studied compounds: A. acesulfame potassium (ACE), B. benzoate acid (BEN), C. sorbic acid (SOR), D. sodium saccharin (SAC), E. tartrazine (TAR), F. caffeine (CAF), G. sunset yellow FCF (SUN), and H. aspartame (ASP).
