# Supplementary material for: Development and Validation of HPLC-DAD Method for Simultaneous Determination of Seven Food Additives and Caffeine in Powdered Drinks
Source: Foods. 2020 Aug 13;9(8):1119. doi: 10.3390/foods9081119 (PMC7466259; doi:10.3390/foods9081119)
Supplement: Supplementary file 1 [file foods-09-01119-s001.pdf]

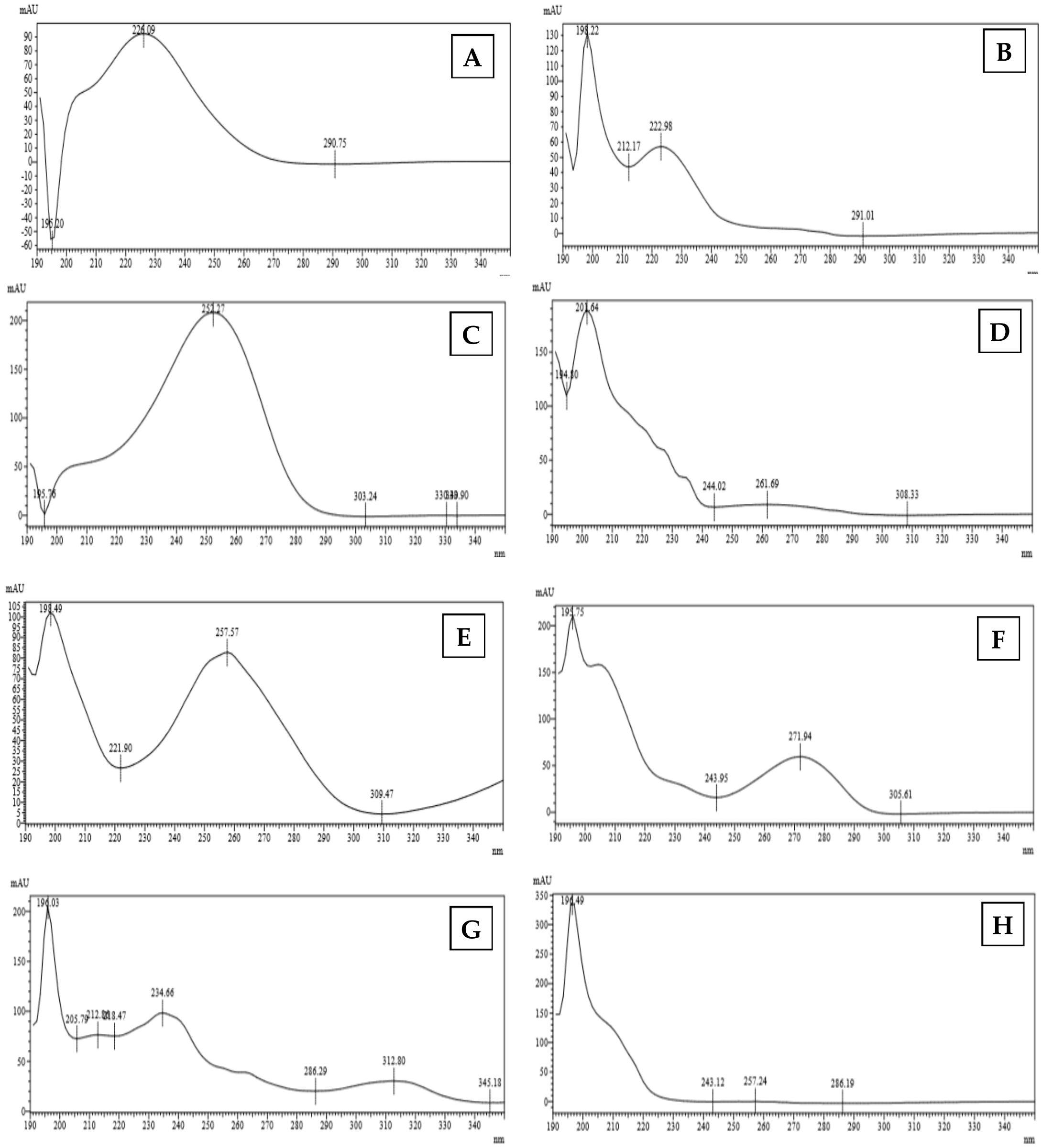

Supplementary Figure S1. Spectra of the studied compounds: A. acesulfame potassium (ACE), B. benzoate acid (BEN), C. sorbic acid (SOR), D. sodium saccharin (SAC), E. tartrazine (TAR), F. caffeine (CAF), G. sunset yellow FCF (SUN), and H. aspartame (ASP).
